# A Holistic Approach to Marine Eco-Systems Biology

**DOI:** 10.1371/journal.pbio.1001177

**Published:** 2011-10-18

**Authors:** Eric Karsenti, Silvia G. Acinas, Peer Bork, Chris Bowler, Colomban De Vargas, Jeroen Raes, Matthew Sullivan, Detlev Arendt, Francesca Benzoni, Jean-Michel Claverie, Mick Follows, Gaby Gorsky, Pascal Hingamp, Daniele Iudicone, Olivier Jaillon, Stefanie Kandels-Lewis, Uros Krzic, Fabrice Not, Hiroyuki Ogata, Stéphane Pesant, Emmanuel Georges Reynaud, Christian Sardet, Michael E. Sieracki, Sabrina Speich, Didier Velayoudon, Jean Weissenbach, Patrick Wincker, the Tara Oceans Consortium

**Affiliations:** 1 European Molecular Biology Laboratory (EMBL), Heidelberg, Germany; 2 Institut de Ciències del Mar (ICM), Consejo Superior de Investigaciones Científicas (CSIC), Barcelona, Spain; 3 CNRS, Paris, France; 4 Institut de Biologie de l'Ecole Normale Supérieure (IBENS), Paris, France; 5 Station Biologique de Roscoff (SBR), Evolution du Plancton et Paleo Oceans (EPPO), Roscoff, France; 6 Université Pierre et Marie Curie (UPMC), Paris, France; 7 VIB, Brussels, Belgium; 8 Vrije Universiteit Brussel, Brussels, Belgium; 9 University of Arizona, Tucson, Arizona, United States of America; 10 University of Milano-Bicocca, Milan, Italy; 11 Aix-Marseille Université, Marseilles, France; 12 MIT, Cambridge, Massachusetts, United States of America; 13 Laboratoire d'Oceanographie de Villefranche (LOV), Villefranche-sur-Mer, France; 14 Stazione Zoologica Anton Dohrn, Naples, Italy; 15 Commissariat à l'énergie atomique et aux énergies alternatives (CEA), Genoscope, Evry, France; 16 Universitaet Bremen, Bremen, Germany; 17 MARUM Center for Marine Environmental Sciences, Bremen, Germany; 18 University College Dublin, Dublin, Ireland; 19 BioDev, Villefranche-sur-Mer, France; 20 Bigelow Laboratory for Ocean Sciences, West Boothbay Harbor, Maine, United States of America; 21 Laboratoire de Physique des Océans (LPO), CNRS/Institut français de recherche pour l'exploitation de la mer (IFREMER)/Institut de Recherche pour le Développement (IRD)/Université de Bretagne Occidentale (UBO), Brest, France; 22 DVIPC, Paris, France

## Abstract

The structure, robustness, and dynamics of ocean plankton ecosystems remain poorly understood due to sampling, analysis, and computational limitations. The Tara Oceans consortium organizes expeditions to help fill this gap at the global level.

## Introduction

With biology becoming quantitative, systems-level studies can now be performed at spatial scales ranging from molecules to ecosystems. Biological data generated consistently across scales can be integrated with physico-chemical contextual data for a truly holistic approach, with a profound impact on our understanding of life [1]–[5]. Marine ecosystems are crucial in the regulation of Earth's biogeochemical cycles and climate [6],[7]. Yet their organization, evolution, and dynamics remain poorly understood [8],[9]. The *Tara* Oceans project was launched in September 2009 for a 3-year study of the global ocean ecosystem aboard the ship *Tara.* A unique sampling programme encompassing optical and genomic methods to describe viruses, bacteria, archaea, protists, and metazoans in their physico-chemical environment has been implemented. Starting as a grassroots initiative of a few scientists, the project has grown into a global consortium of over 100 specialists from diverse disciplines, including oceanography, microbial ecology, genomics, molecular, cellular, and systems biology, taxonomy, bioinformatics, data management, and ecosystem modeling. This multidisciplinary community aims to generate systematic, open access datasets usable for probing the morphological and molecular makeup, diversity, evolution, ecology, and global impacts of plankton on the Earth system.

Viruses, bacteria, archaea, protists, and planktonic metazoans form the bulk of biomass throughout the oceans and drive the global biogeochemical cycles that regulate the Earth system [6],[9],[10]. For instance, marine microbes produce nearly as much oxygen through primary production as land plants [11]. This system is driven by a complex ecological network of autotrophic, heterotrophic, and mixotrophic organisms, where trophodynamics and biogeochemical interdependencies are determining factors for primary production rates in marine systems. In addition, ocean viruses modulate primary production by inducing organism mortality and by encoding core photosynthesis genes that are expressed during infection [12]–[14]. Therefore, only an ecosystem-wide approach, from viruses to metazoans, will enable us to start disentangling the functioning of the Earth system. This approach ranges from mapping organismal diversity across scales spanning five orders of magnitude to developing empirical datasets that inform conceptual models about the complex interplay between organisms driving fluxes of energy, biogeochemical, and molecular “currencies” in ocean ecosystems [15].

A global-scale study of morphological, genetic, and functional biodiversity of plankton organisms in relation to the changing physico-chemical parameters of the oceans [8],[16]–[18] is now critical to understanding and managing our fragile oceans. Specifically, such a dataset will improve our understanding of the principles governing marine ecosystems and the evolution of life in the ocean, thus enhancing our capacity of assessing ecosystem services and enabling a better prediction of fish stock distribution and impacts of global climate variations [19]. Planktonic organisms are also an enormous but largely untapped source [8],[20] of bio-active compounds for the pharmaceutical, food, and cosmetics industries, as well as metabolic pathways that may provision our future energy needs [21]. In this context, the *Tara* Oceans consortium was founded, which embarked on a 3-year research cruise across the worlds' oceans.

**Figure 1:**
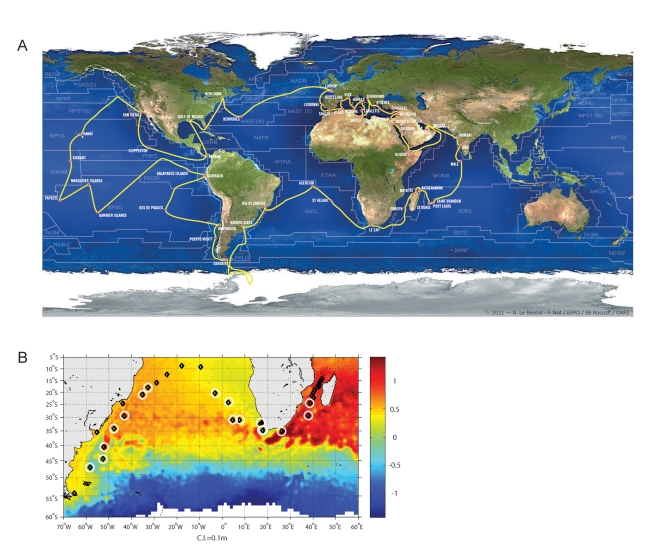
The *Tara* Oceans cruise. (A) Route of the *Tara* Oceans expedition. Sampling stations from surface to 1,000 m are carried out between ports of call guided by satellite data about the basin to sub-mesoscale structures. (B) *Tara* Oceans sampling sites in the Mozambique Channel and South Atlantic. The images show near real time sea surface height (SSH) from satellite. Each sampling station is indicated by a black diamond; those that are currently being targeted for priority studies are encircled by a white halo. The altimetry data is from September 16, 2011, when *Tara* was sampling inside an Agulhas ring. Several other rings are also apparent in the figure, as is the Malvinas Current off the Argentinean coast that injects cold Antarctic water into the resident waters.

*Tara* Oceans is not the first group to explore global ocean biodiversity. For example, previous global initiatives include satellite-based chlorophyll measurements, the Census of Marine Life, long-term observation sites, and arrays of remote sensors on floats that provide physical, chemical, and biological data [15]. Other global genomics studies have been launched, e.g., Global Ocean Sampling (GOS) expedition [22] and the Earth Microbiome [23] project, as well as integrative projects focusing on specific biomes (e.g., Malaspina, http://www.expedicionmalaspina.es/). However, *Tara* Oceans takes such investigations one step further by integrating the genetic, morphological, and functional diversity in its environmental context at global ocean scale and at multiple depths (Figure 1), from viruses to fish larvae. While such a “study it all” approach is not novel (e.g., NSF Long Term Ecological Research sites), it has remained science fiction until technology and informatics became enabling. Now, high throughput sequencing, quantitative imaging methods, dedicated bio-informatics and modeling tools are poised to assess the complexity of the global ocean systems. To achieve such integration, *Tara* Oceans is driven by researchers with expertise in biological and physical oceanography, ecology, microbiology, systematics, molecular, cellular and systems biology, bioinformatics, data management, and modeling.

Pragmatically, to accomplish such an ambitious goal, *Tara *Oceans consortium scientists have necessarily been intimately involved in every aspect of the expedition. This includes planning, preparation, and running of the on-board sampling protocols, as well as the development of sample analysis and bioinformatics pipelines, data management, and modeling projects. This involvement ensures a coherent worldwide data collection and analysis strategy, which is reinforced through regular workshops. The consortium has an open access policy concerning the data that will be made available to the scientific community as soon as possible after validation. Finally, a significant outreach effort is made to show life on board of *Tara* as well as translate results to the broader public (see http://oceans.taraexpeditions.org/ and http://www.planktonchronicles.org).

## The Expedition and Sampling Strategy

**Figure 2:**
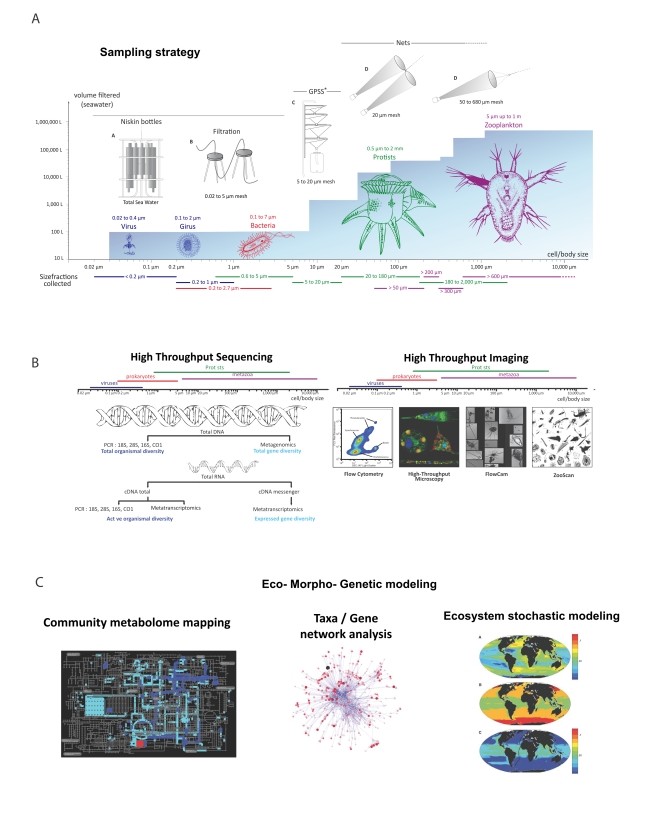
The *Tara* Oceans model. (A) Methods for sampling organisms by size classes and abundance. The blue background indicates the filtered volume required to obtain sufficient organism numbers for analysis. Actual volumes from which organisms are sampled are always recorded. (B) Methods for analyzing samples. Data on the right are from *Tara* Oceans sampling stations. (C) Models that will benefit from *Tara* Oceans data. High throughput genome sequencing and quantitative image analysis provide evolution, metabolic, and interaction data to build community metabolome maps, taxa/gene networks, and spatial ecosystem models.

To collect and fractionate plankton communities on the basis of organism size, spanning five orders of magnitude (Figure 2A), the sampling combines traditional (Niskin bottles and plankton net tows) and novel methods (e.g., a gravity-driven plankton sieve and chemistry-based concentration of viruses [24]) that feed into analytical pipelines appropriate for each size class. These analyses range from immediate visualization and quantification on-board *Tara* with a diversity of imaging tools, to collection and preservation of samples for genomic and morphological analysis back on land.

Given the global nature of the expedition and spatial heterogeneity in the oceans, a concern is the “snapshot” sampling strategy employed here at a single time point and at relatively sparse stations relative to the global ocean, which represents the inherent challenge of global ocean studies. In *Tara* Oceans we leverage near real-time remote sensing and other data to locate oceanographically interesting features (e.g., eddies, fronts, upwellings) and strengthen ecosystem comparisons (Figures 1B and 2A). The vast ocean basins are relatively homogeneous on seasonal to decadal time scales, whereas smaller-scale systems are more dynamic. For example, the Agulhas leakage system (Figure 1B) transports water from the Indian to the Atlantic Ocean and across to South America leaving heterogeneous ocean features behind that persist for weeks to months [25]. The Agulhas system represents an ideal case for applying the *Tara* Oceans near real-time sampling strategy and downstream analysis pipeline to deeply characterize the biology of these ecosystems. Through systematic study of such heterogenous systems coupled to broader “normal” ocean sampling, we hope to unveil the rules that govern the structure and dynamics of ocean ecosystems and to extrapolate such local observations to develop basin-scale process models as predictive tools [26]. Undoubtedly, such measurements and predictions will provide a starting point for hypothesis testing by more focused, follow-up campaigns.

## The *Tara* Oceans Integrated Pipeline: Towards Eco-Systems Biology

The scientific programme of *Tara* Oceans requires an integrated pipeline that combines semi- or fully automated data acquisition methods, new bioinformatics tools, and standardized data organisation (Figure 2C). The high throughput imaging platform includes instruments tuned to organisms of particular size classes. They include (i) on-board and on-land flow cytometers to monitor virus particles, bacteria, and small protists, (ii) on-land digital and confocal microscopy for detailed 2D/3D imaging of cells within the 5–20-µm range, (iii) on-board and on-land FlowCams and ZooScans for quantitative recognition of organisms ranging from 20 µm to a few cm, light sheet and confocal microscopes for 3D imaging, and (iv) on-land electron microscopes for detailed ultrastructural analyses of small protists and viruses. In parallel, we use high throughput sequencing methods to obtain both deep phylogenetic rDNA/rRNA tag data and metagenomic and metatranscriptomic functional profiles from size fractions covering the entire plankton community from viruses to fish larvae (Figure 2B and 2C). To bring all these data together for analyses, we leverage recently developed, dedicated computational approaches [27]. In addition, *Tara* Oceans is archiving meteorological, oceanographic, biogeochemical, and plankton morphology data in the PANGAEA database (http://www.pangaea.de/), linking to larger European and international data infrastructures. Thus, *Tara* Oceans can visualize, quantify, and genetically characterize ocean biodiversity within entire plankton ecosystems, as well as find patterns across unprecedentedly comprehensive data types.

With such a powerful dataset and toolkit, we anticipate testing the predictions of biodiversity hotspots from stochastic modelling [28]–[31], as well as mapping functional gene ecology and activities throughout the world's oceans. This process has been initiated, predominantly for 0.1–0.8-µm-sized surface ocean microbes, using data provided by the GOS expedition [22],[27],[32],[33], but will be dramatically extended here by sampling throughout the water column, across organismal size scales and beyond metagenomic sequencing, tightly coupled to global ocean-modelling efforts.

These data will transform our ability to link species diversity and metabolic potential/activity to environmental conditions and ecosystem outputs and promises to lead to the discovery of emergent ecological principles (Figure 2C). They will allow a deeper understanding of biodiversity gradients within and among systems and contrasting environments. We also anticipate establishing rules governing the self-organization of organism networks (e.g., to address which types of viruses, bacteria, protists, and zooplankton live together in a given environment) [16],[27], and develop predictions about how these rules and communities will be affected by a changing environment.

In summary, the *Tara* Oceans project leverages powerful new technologies and analytical tools to develop the first planetary-scale data collection effort that links biogeography with ecology, genetics, and morphology. Guided by the cross-disciplinary philosophy the pay-offs can be immense, considering the massive number of samples and data that have been collected, archived, and interconnected for scientific study, only half-way through the expedition. A lesson from this project is that, when it comes to addressing broad and complex issues of general interest to mankind, competition between scientists may not be the best model. The *Tara* Oceans project is a pioneering enterprise towards a truly worldwide, systems-level characterization of the largest and most fundamental ecosystem on our planet.

## References

[1] Karsenti E (2008). Self-organization in cell biology: a brief history.. Nat Rev Mol Cell Biol.

[2] Keurentjes J. J, Angenent G. C, Dicke M, Santos V. A, Molenaar J (2011). Redefining plant systems biology: from cell to ecosystem.. Trends Plant Sci.

[3] Krakauer D. C, Collins J. P, Erwin D, Flack J. C, Fontana W (2011). The challenges and scope of theoretical biology.. J Theor Biol.

[4] Nédélec F, Surrey T, Karsenti E (2003). Self-organisation and forces in the microtubule cytoskeleton.. Curr Opon Cell Biol.

[5] Raes J, Bork P (2008). Molecular eco-systems biology: towards an understanding of community function.. Nat Rev Microbiol.

[6] Arrigo K. R (2005). Marine microorganisms and global nutrient cycles.. Nature.

[7] Falkowski P. G, Katz M. E, Knoll A. H, Quigg A, Raven J. A (2004). The evolution of modern eukaryotic phytoplankton.. Science.

[8] Bowler C, Karl D. M, Colwell R. R (2009). Microbial oceanography in a sea of opportunity.. Nature.

[9] Falkowski P. G, Fenchel T, Delong E. F (2008). The microbial engines that drive Earth's biogeochemical cycles.. Science.

[10] Karl D. M (2007). Microbial oceanography: paradigms, processes and promise.. Nat Rev Microbiol.

[11] Field C. B, Behrenfeld M. J, Randerson J. T, Falkowski P (1998). Primary production of the biosphere: integrating terrestrial and oceanic components.. Science.

[12] Lindell D, Jaffe J. D, Johnson Z. I, Church G. M, Chisholm S. W (2005). Photosynthesis genes in marine viruses yield proteins during host infection.. Nature.

[13] Sharon I, Tzahor S, Williamson S, Shmoish M, Man-Aharonovich D (2007). Viral photosynthetic reaction center genes and transcripts in the marine environment.. ISME J.

[14] Sullivan M. B, Lindell D, Lee J, Thompson L. R, Bielawski J. P (2006). Prevalence and evolution of core photosystem II genes in marine cyanobacterial viruses and their hosts.. PLoS Biol.

[15] Lupp C (2009). Microbial oceanography.. Nature.

[16] Fuhrman J. A (2009). Microbial community structure and its functional implications.. Nature.

[17] Irigoien X, Huisman J, Harris R. P (2004). Global biodiversity patterns of marine phytoplankton and zooplankton.. Nature.

[18] Rohwer F, Thurber R. V (2009). Viruses manipulate the marine environment.. Nature.

[19] Smith J, Wigginton N, Ash C, Fahrenkamp-Uppenbrink J, Pennisi E (2010). Changing oceans. Introduction.. Science.

[20] Sarmento H, Montoya J. M, Vázquez-Domínguez E, Vaqué D, Gasol J. M (2010). Warming effects on marine microbial food web processes: how far can we go when it comes to predictions?. Philos Trans Royal Soc London B Biol Sci.

[21] Arnaud-Haond S, Arrieta J. M, Duarte C. M (2011). Global genetic resources. Marine biodiversity and gene patents.. Science.

[22] Rusch D. B, Halpern A. L, Sutton G, Heidelberg K. B, Williamson S (2007). The Sorcerer II Global Ocean Sampling expedition: northwest Atlantic through eastern tropical Pacific.. PLoS Biol.

[23] Gilbert J. A, Meyer F, Antonopoulos D, Balaji P, Brown C. T (2010). Meeting report: the terabase metagenomics workshop and the vision of an Earth microbiome project.. Stand Genomic Sci.

[24] John S. G, Mendez C. B, Deng L, Poulos B, Kauffman A. K. M (2010). A simple and efficient method for concentration of ocean viruses by chemical flocculation.. Environ Microbiol Rep.

[25] Beal L. M, De Ruijter W. P. M, Biastoch A, Zahn R (2011). On the role of the Agulhas system in ocean circulation and climate.. Nature.

[26] Follows M. J, Dutkiewicz S, Grant S, Chisholm S. W (2007). Emergent biogeography of microbial communities in a model ocean.. Science.

[27] Raes J, Letunic I, Yamada T, Jensen L. J, Bork P (2011). Toward molecular trait-based ecology through integration of biogeochemical, geographical and metagenomic data.. Mol Syst Biol.

[28] d'Ovidio F, De Monte S, Alvain S, Dandonneau Y, Lévy M (2010). Fluid dynamical niches of phytoplankton types.. Proc Natl Acad Sci U S A.

[29] Tittensor D. P, Mora C, Jetz W, Lotze H. K, Ricard D (2010). Global patterns and predictors of marine biodiversity across taxa.. Nature.

[30] Barton A. D, Dutkiewicz S, Flierl G, Bragg J, Follows M. J (2010). Patterns of diversity in marine phytoplankton.. Science.

[31] Bragg J. G, Dutkiewicz S, Jahn O, Follows M. J, Chisholm S. W (2010). Modeling selective pressures on phytoplankton in the global ocean.. PLoS ONE.

[32] Gianoulis T. A, Raes J, Patel P. V, Bjornson R, Korbel J. O (2009). Quantifying environmental adaptation of metabolic pathways in metagenomics.. Proc Natl Acad Sci U S A.

[33] Temperton B, Gilbert J. A, Quinn J. P, McGrath J. W (2011). Novel analysis of oceanic surface water metagenomes suggests importance of polyphosphate metabolism in oligotrophic environments.. PLoS ONE.

